# A rare cause of infant facial paralysis: atypical teratoid rhabdoid tumour located in the cerebellopontine angle

**DOI:** 10.1186/s40064-015-1526-1

**Published:** 2015-11-25

**Authors:** Mehmet Öztürk, Ahmet Siğirci, Neşe Karadağ

**Affiliations:** Diyarbakır Children’s Hospital, 21100 Diyarbakır, Turkey; Faculty of Medicine, Inonu University, Elazig road, 15 km, Malatya, Turkey

**Keywords:** Facial palsy, Children, Atypical teratoid rhabdoid tumour, Cerebellopontine angle

## Abstract

Atypical teratoid rhabdoid tumour (ATRT) is a rare malignant tumour of the central nervous system with embryonal roots. The majority are seen in early childhood and location is often in the posterior fossa. Surgery, radiotherapy and chemotherapy are used in treatment. Knowledge of the localisation of the mass preoperatively is necessary for direction of the chemoradiotherapy and sufficient resection in surgery. Differentiation from other brain tumours is important because of poor prognosis and differences in treatment. In this paper it was aimed to present the clinical and radiological findings of an ATRT located in the cerebellopontine angle, which occurred with facial paralysis.

## Background

In the differential diagnosis of infants with acute facial paralysis, the possibility of ATRT should be considered.

## Case presentation

A 1-year old male infant was brought to the Paediatric Emergency Department with the acute development of left-side facial paralysis. With an initial diagnosis of potential intracranial mass, contrast brain computed tomography (CT) and magnetic resonance imaging (MRI) tests were applied.

**Fig. 1:**
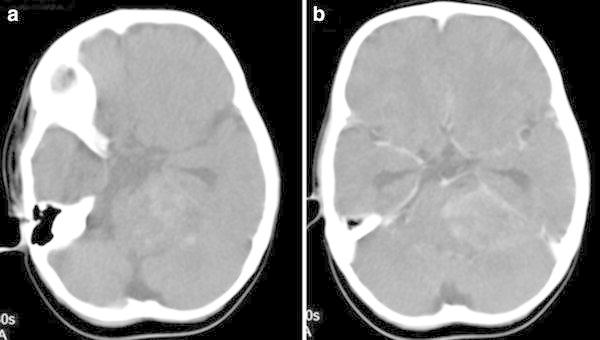
**a, b** On CT images without contrast, isodense with the cerebellar parenchyma in the left CPA, amorphous calcifications including an extra-axial, dural-based mass lesion 4 × 3 cm in size, with heterogenous contrast after the application of IV contrast dye. Time there is concomitant calcification, bleeding and oedema of the surrounding

On the brain CT images, an extra-axial, dural-based mass lesion was observed 4 × 3 cm in size, with heterogenous contrast, time there is concomitant calcification, bleeding and oedema of the surrounding, which was isodense with the cerebellar parenchyma in the CPA (Fig. 1a, b).

**Fig. 2:**
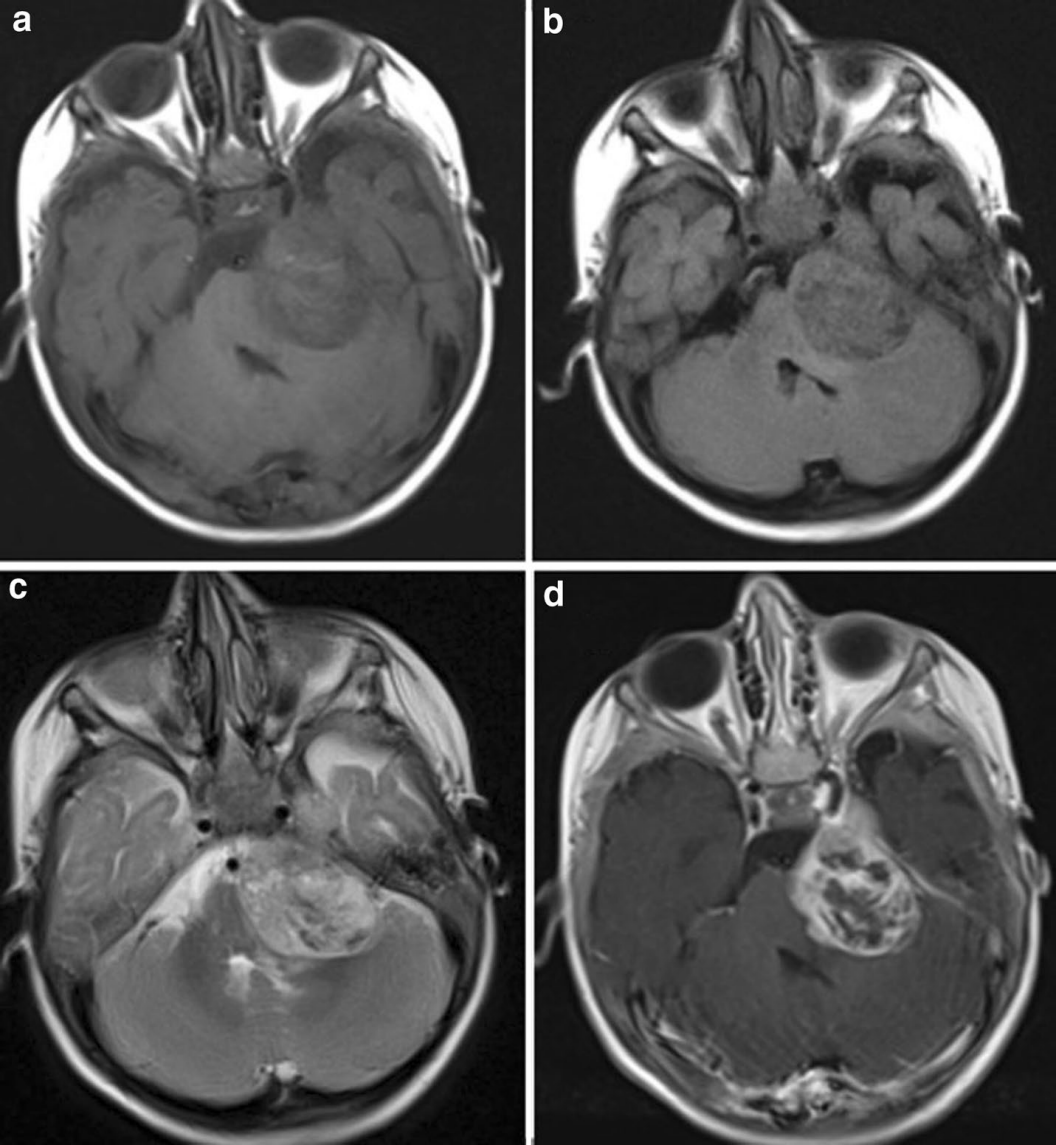
**a–d** On the brain MRI, a dural-based, extra-axial mass lesion was observed on the left CPA, 4 × 3 cm in size, with heterogenous contrast after the application of IV contrast dye, which was heterogenous-hypointense on T1W and FLAIR sequences, heterogenous hyperintense on T2W, the mass appears to enter the cavernous sinus via Meckle’s cave. Time there is concomitant calcification, bleeding and oedema of the surrounding

On the brain MRI, a dural-based, extra-axial mass lesion was observed on the left CPA, 4 × 3 cm in size, with heterogenous contrast, which was heterogenous-hypointense on T1W and FLAIR sequences, heterogenous-hyperintense on T2W. The mass appears to enter the cavernous sinus via Meckle’s cave. Time there is concomitant calcification, bleeding and oedema of the surrounding (Fig. 2a–d).

No spread was determined to other regions of the brain or to the spinal cord.

**Fig. 3:**
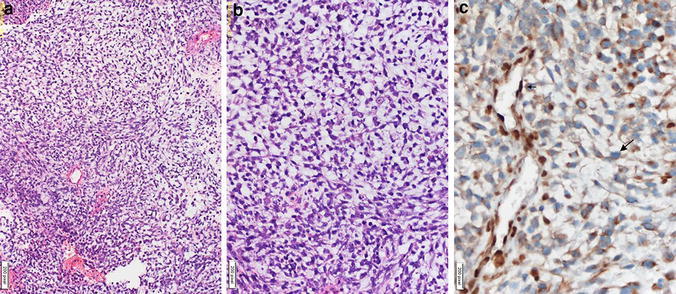
**a–c** Immunohistochemical staining of pathological specimen showing AT/RT. Hypercellular spindle and consists of a round core tumor cells (**a**, **b**), nuclear INI-1 negativity on tumor cells (*large arrow*), endothelial nuclear staining positive internal control (*small arrow*) (**c**)

The pathology diagnosis after surgery reported the mass as ATRT (Fig. 3a–c).

### Differential diagnosis

In the differential diagnosis of tumours located in the CPA in infants, medulloblastoma, schwannoma, ependymoma and astrocytoma should be considered.

### Treatment

With an initial diagnosis of a mass located in the CPA, the patient was admitted for surgery. As the mass had invaded the cavernous sinus via Meckle’s cave, it could not be completely resected.

### Outcome and follow-up

At 1 month postoperatively the patient developed a pulmonary infection and due to a worsening of the general condition, he died while under observation in the intensive care unit.

## Discussion

The most common reason for facial paralysis in children is Bell’s Palsy followed by tumoural lesions. The most frequently seen tumour is cerebellar astrocytoma (Shih et al. 2009). ATRT is a rare embryological tumour of the central nervous system and is usually seen in early childhood. It constitutes 2–3 % of paediatric brain tumours. The majority are diagnosed before the age of 3 years (Lee et al. 2004). Generally the location is in the posterior fossa and less frequently, cerebral hemisphere, pineal region and spinal cord involvement is seen (Hilden et al. 1998). Location in the CPA is extremely rare (Siu et al. 2014).

Together with clinically non-specific symptoms of paediatric CPA located tumours such as macrocephaly, lethargy, vomiting and headache, specific symptoms may be seen such as strabismus, facial paralysis and double vision which shows double involvement in the head (Gyure 2005). Of ATRT with a posterior fossa location, 46 % emerge with cranial nerve palsies (Chen et al. 2005).

The imaging findings of previously reported cases in literature have been similarly non-specific. Generally, the mass is in heterogenous form within cystic necrotic areas. On CT, the solid component is of moderate or high density compared to the grey matter. Most of the time there is concomitant calcification, bleeding and oedema of the surrounding (Meyers et al. 2006). In the current case, without contrast on CT a lesion was observed isodense with the grey matter and after IV contrast, as a contrasting solid component as a mass lesion of heterogenous density filling the left cerebellopontine angle.

ATRT located in the CPA have similar imaging features as lesions in other areas of the brain. On T1W images on MRI, a cystic-necrotic area is observed in a heterogenous signal due to haemorrhage and calcification. On T2W images, there is heterogenous intensity at mild-moderate and high signals. Blood products and calcification are seen as hypointense and the cystic-necrotic areas as hyperintense. On T2W and FLAIR images, the solid component shows evident contrast compared to the grey matter at moderate and high signals (Siu et al. 2014; Meyers et al. 2006; Hanna et al. 1993). In the current case, the MRI findings of the mass were similar to those in literature but no bleeding was determined. Furthermore, it was confirmed during surgery that there was extension from the foramen lacerum to the temporal lobe and adjacent to the left cavernous sinus.

The differential diagnosis of tumours located in the CPA in an infant includes medulloblastoma, schwannoma, ependymoma and astrocytoma (Tsai et al. 2009). According to imaging findings, the most commonly encountered tumour is medulloblastoma. However, medulloblastoma occurs with multiple cranial nerve paralyses which develop over a long time. Facial paralysis alone is extremely rare and is a late-stage symptom (Jaiswal et al. 2004). In the current case, sudden onset unilateral facial paralysis was the only clinical sign.

In an infant with a mass located in the CPA together with acute development of facial paralysis, ATRT must be considered in the differential diagnosis.

## Conclusion

Mass lesions are important in the differential diagnosis in infants that develop acute facial paralysis. ATRT must be included in the mass lesions.

## Abbreviations

ATRT: Atypical teratoid rhabdoid tumour
CT: Computed tomography
MRI: Magnetic resonance imaging
CPA: Cerebellopontine angle

## References

[CR1] Chen ML, McComb JG, Krieger MD (2005). Atypical teratoid/rhabdoid tumors of the central nervous system: management and outcomes. Neurosurg Focus.

[CR2] Gyure KA (2005). Newly defined central nervous system neoplasms. Clin Pathol.

[CR3] Hanna SL, Langston JW, Parham DM, Douglass EC (1993). Primary malignant rhabdoid tumor of the brain: clinical, imaging and pathologic findings. AJNR Am J Neuroradiol.

[CR4] Hilden JM, Watterson J, Longee DC, Moertel CL, Dunn ME, Kurtzberg J (1998). Central nervous system atypical teratoid/rhabdoid tumor: response to intensive therapy and review of literature. J Neurooncol.

[CR5] Jaiswal AK, Mahapatra AK, Sharma MC (2004). Cerebellopointine angle medulloblastoma. J Clin Neurosci.

[CR6] Lee YK, Choi CG, Lee JH (2004). Atypical teratoid/rhabdoid tumor of the cerebellum: report of two infantile cases. ANJR Am J Neuroradiol.

[CR7] Meyers SP, Khademian ZP, Biegel JA, Chuang SH, Korones DN, Zimmerman RA (2006). Primary intracranial atypical teratoid/rhabdoid tumors of infancy and childhood: MRI features and patient outcomes. AJNR Am J Neuroradiol.

[CR8] Shih WH, Tseng FY, Yeh TH, Hsu CJ, Chen YS (2009). Outcomes of facial palsy in children. Acta Otolaryngol.

[CR9] Siu A, Lee M, Rice R, Myseros JS (2014). Association of cerebellopontine angle atypical teratoid/rhabdoid tumors with acute facial nerve palsy in infants. J Neurosurg Pediatr.

[CR10] Tsai MH, Wong AM, Jaing TH, Wang HS, Hsueh C, Wu CT (2009). Treatment of cerebellopontine angle tumors in children: a single institution’s experience. J Pediatr Hematol Oncol.

